# Stealth Properties to Improve Therapeutic Efficacy of Drug Nanocarriers

**DOI:** 10.1155/2013/374252

**Published:** 2013-03-07

**Authors:** Stefano Salmaso, Paolo Caliceti

**Affiliations:** Department of Pharmaceutical and Pharmacological Sciences, University of Padua, Via F. Marzolo 5, 35131 Padova, Italy

## Abstract

Over the last few decades, nanocarriers for drug delivery have emerged as powerful tools with unquestionable potential to improve the therapeutic efficacy of anticancer drugs. Many colloidal drug delivery systems are underdevelopment to ameliorate the site specificity of drug action and reduce the systemic side effects. By virtue of their small size they can be injected intravenously and disposed into the target tissues where they release the drug. Nanocarriers interact massively with the surrounding environment, namely, endothelium vessels as well as cells and blood proteins. Consequently, they are rapidly removed from the circulation mostly by the mononuclear phagocyte system. In order to endow nanosystems with long circulation properties, new technologies aimed at the surface modification of their physicochemical features have been developed. In particular, stealth nanocarriers can be obtained by polymeric coating. In this paper, the basic concept underlining the “stealth” properties of drug nanocarriers, the parameters influencing the polymer coating performance in terms of opsonins/macrophages interaction with the colloid surface, the most commonly used materials for the coating process and the outcomes of this peculiar procedure are thoroughly discussed.

## 1. Introduction

Cancer is a leading cause of death worldwide as accounted for 7.6 million deaths (around 13% of all deaths) in 2008 (source: WHO Fact sheet N°297 February 2012). About 70% of all cancer deaths occurred in low- and middle-income countries. Deaths caused by cancer are forecasted to rise to over 13.1 millions in 2030 (Globocan, 2008, IARC, 2010). 

Nevertheless, over the past few decades, significant advances have been made in fundamental cancer biology, allowing for remarkable improvements in diagnosis and therapy for cancer. Beside the development of new drugs with potent and selective activities, nanotechnology offers novel opportunities to cancer fighting by providing adequate tools for early detection and personalized treatments. 

Over the last decades, a number of different long circulating vehicles have been developed for theranostic purposes. These carriers are in the nanometer range size and most of them have been intended for the delivery of anticancer drugs to tissues affected by this pathology. 

The aim of this paper is to examine the features of “stealth” long circulating nanocarriers and the pharmacokinetic outcomes of stealthiness, and it will showcase the most investigated approaches yielding prolonged circulation of surface-engineered nanocarriers. 

## 2. The Opsonisation Process

The selective and controlled delivery of anticancer drugs to disease tissues is a requisite to prevent systemic toxicity, enhance the pharmacological profiles, and improve the patient compliance, which in turn provide for amelioration of antitumour therapy. 

Due to the leaky vasculature and low lymph drainage, solid tumours present erratic fluid and molecular transport dynamics. These features can yield specific accumulation of colloidal anticancer drug delivery systems into the tumour tissue by enhanced permeation and retention (EPR) effect [1]. However, in order to exploit the physiopathological and anatomical peculiarities of the tumour tissues, the nanovehicles need prolonged circulation in the bloodstream, ideally over 6 hours [2]. 

The permanence in the bloodstream of nanovehicles is strongly affected by physical interactions with specific blood circulating components, opsonins. These components prevalently include complement proteins such as C3, C4, and C5, laminin, fibronectin, C-reactive protein, type I collagen, and immunoglobulins [3].

Surface opsonisation promotes the removal of particles from the circulation within seconds to minutes through the mononuclear phagocytic system (MPS), also known as reticuloendothelial system (RES), and by Kupffer cells, phagocytic macrophages permanently located in the liver [4]. The natural role of opsonins is to promote the bacteria and viruses approach by the phagocytic cells, both systems having the same negative charge that inhibits the interaction between bacteria/viruses and the phagocytes due to charge repulsion [5]. After bacteria and virus coating, opsonins undergo conformational rearrangements that induce the biorecognition by phagocytes through specific membrane receptors. The xenoparticle opsonisation by complement proteins, over 30 soluble and membrane-bound proteins, induces the complement activation through a cascade of physiological events. The opsonisation finally promotes the removal process by phagocytes [4].

**Figure 1:**
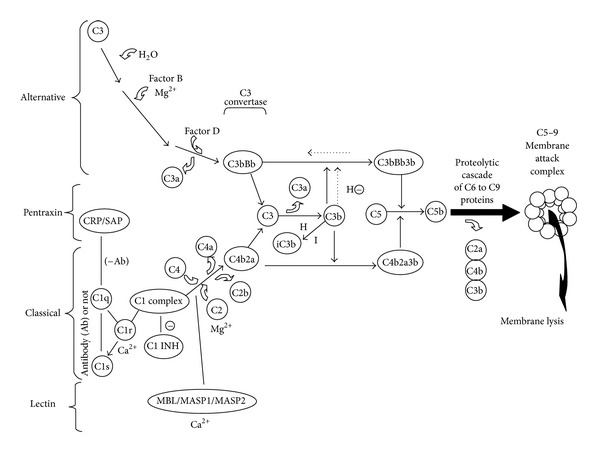
Schematic representation of the different activation pathways of the complement system. (Reprinted with permission from *Biomaterials*, 2006, 27, 4356–4373. Copyright ©2006 Elsevier Ltd.)

The complement is a key component of innate immunity that naturally monitors host invaders through three distinct activation pathways described in Figure 1 [6]. 

The classical pathway is activated after the fixation of C1q proteins to antibodies or to C1q receptors on the cell surface. The alternative pathway is spontaneously activated by the binding of C3 fragments to the surface of the pathogen. The lectin pathway is activated by the binding of mannose-binding lectin on mannose contained on the surface corona of bacteria and viruses. Although a few hypotheses have been proposed to explain the existence of supplementary activation pathways, they have not been fully elucidated. 

Regardless of the activation pathway, the enzymatic cascade of the complement activation leads to the formation of a common enzyme, C3 convertase, which cleaves the central protein of the complement system, the third component C3 [7]. The fragment C3b of C3 is the crucial active component that triggers the cleavage of a variety of complement proteins (C5–C9). The assembly of these proteins contributes to the formation of the membrane attack complex (MAC) that is able to destabilize bacteria, viruses, and nanocarriers for drug delivery. C3b and its inactive fragment iC3b can be recognised by specific receptors on phagocytic cells leading to the engulfing of opsonised particles and their removal from the bloodstream. 

Additionally, the complement activation triggers a cascade of inflammatory and adverse complex reactions, named complement activation-related pseudoallergy (CARPA), that reflect in symptoms of transient cardiopulmonary distress. These effects have been detailed by the literature [8–11].

The complement system is also finely regulated by the presence of inhibitor proteins such as C1 INH, Factor I and H [12]. 

Even though the natural role of opsonisation is directed to the body protection from xenogeneic nanosystems, this process promotes the removal of circulating drug nanocarriers. This represents a major obstacle to achieve adequate systemic and local therapeutic drug concentrations. 

### 2.1. Steric Shielding and Stealth Properties of Nanocarriers

In the bloodstream, opsonins interact with nanoparticles by van der Waals, electrostatic, ionic, and hydrophobic/hydrophilic forces. Therefore, the surface features of the nanocarriers have a key role in the opsonisation process. Hydrophobic and charged particles undergo higher opsonisation as compared to hydrophilic and neutrally charged particles [13–16]. 

In the last decades, different theories have been attempted to describe the pharmacokinetic profiles of nanosized drug delivery systems, namely, liposomes and polymeric nanoparticles. It is now recognised that long circulating nanocarriers, “stealth” systems, can be obtained by surface coating with hydrophilic polymers that prevent the opsonisation process [17–19]. The consequence of avoiding opsonisation is the prolongation of the liposome and particle permanence in the bloodstream from few seconds to several hours [17, 20, 21]. 

Peppas described the effect of the hydrophilic polymer shell on nanoparticle surface in terms of elastic forces. He focused the attention on PEG that is the most representative of the materials used to produce stealth nanocarriers. According to their hydrophilic and flexible nature, the PEG chains can acquire an extended conformation on particle surface. Opsonins attracted to the particle surface compress the extended PEG chains that shift to a more condensed and higher energy conformation. As a consequence, the repulsive forces counterbalance the attractive forces between opsonins and the particle surface [22]. 

At low polymer density on the particle surface, when the polymer chains cannot interact with the surrounding chains and may freely collapse on the surface, the polymer chains provide for steric repulsion at a distance *h* according to the equation

(1) $$\begin{array}{c} F_{\text{st}}^{m}=\frac{\left(kT\right)}{\left(D^{2}h_{c}\right){\left(h_{c}/h\right)}^{8/3}}. \end{array}$$

In the equation *F*_st_^*m*^is the steric repulsive force referred to the “mushroom” model (*m*), *h*_*c*_ is the extension of a polymer above the surface = *Na*(*a*/*D*)^2/3^, *D* is the average distance between adjacent grafting points, *a* is the size of the segment, and *N* is the degree of polymerization. 

At high polymer densities, the polymer chains extend and interact with each other exerting the steric repulsive force *F*_st_^br^ referred to the “brush” model (br):

(2) $$\begin{array}{c} F_{\text{st}}^{\text{br}}=\frac{\left(kT\right)}{D^{3}\left[{\left(h_{c}/h\right)}^{9/4}-{\left(h/h_{c}\right)}^{3/4}\right]}. \end{array}$$

These equations describe repulsive phenomena occurring on flat surfaces. However, they can be properly elaborated to gain information about repulsive steric barriers endowed by adsorbed polymers on curved surfaces of stealth nanoparticles [23].

### 2.2. Polymers Used to Coat Nanocarriers

Long circulating nanocarriers are usually obtained by polymer surface coating that endows systems with stealth properties [24]. In drug delivery, the term “stealth,” translated from the “low observable technology” applied to military tactics, refers to nanovehicles that are invisible to the biological system involved in clearance of particle from the bloodstream, namely, RES and Kupffer cells. 

So far, many efforts have been done to yield stealth products by modification of the surface properties of nanocarriers with polymers that prevent opsonin interactions [25] and subsequent phagocyte clearance [26–28]. 

The polymers used to confer stealth properties to nanoparticles and nanovesicles have few basic common features: high flexibility and high hydrophilicity. Either natural and semisynthetic polysaccharides or synthetic polymers have been used for these purposes. Dextran (Dex), polysialic acid (PSA), hyaluronic acid (HA), chitosan (CH), and heparin are the most used natural polysaccharides. Synthetic polymers include polyvinyl pyrrolidone (PVP), polyvinyl alcohol (PVA), polyacrylamide (Pam), poly(ethylene glycol) (PEG), and PEG-based copolymers such as poloxamers, poloxamines, and polysorbates. 

#### 2.2.1. PEG

Poly(ethylene glycol) (PEG) is the polymer of choice to produce stealth nanocarriers. This neutral, flexible, and hydrophilic material can in fact properly produce surface barrier layers that reduce the adhesion of opsonins present in the blood serum on the nanoparticles making them “invisible” to phagocytic cells. The protein repulsion operated by PEG was also visualized by freeze-fracture transmission electron microscopy (TEM) [29].

A few physical protocols have been adopted to coat nanoparticle with PEG [22], even though these procedures entail the risk of polymer desorption in the blood with consequent loss of the beneficial contribution of the polymer [30]. In order to overcome this problem, covalent PEG conjugation protocols have been developed [31, 32]. Biodegradable nanoparticles with PEG covalently bound to the surface have been produced using PEG derivatives of poly(lactic acid), poly(lactic acid-*co*-glycolic acid) [33], or poly(alkylcyanoacrylates) [34]. The nanoparticles are prepared by emulsion, precipitation, or dispersion protocols in aqueous media. These procedures allow for the PEG orientation toward the water phase, while the biodegradable hydrophobic polymer fraction is physically entangled in the inner nanoparticle matrix [22]. Alternatively, PEG chains may be covalently conjugated to preformed nanoparticles through surface functional groups [35, 36]. 

#### 2.2.2. Poloxamine and Poloxamer

Poloxamines (Tetronics) and poloxamers (Pluronics) are amphiphilic block copolymers consisting of hydrophilic blocks of ethylene oxide (EO) and hydrophobic blocks of propylene oxide (PO) monomer units. Poloxamers are a-b-a type triblock copolymers (PEO-PPO-PEO) while poloxamines are tetrablock copolymers of PEO-PPO connected through ethylenediamine bridges [(PEO-PPO)_2_–N–CH_2_–CH_2_–N–(PPO-PEO)_2_] [37–39].

These polymers can be physically adsorbed on the nanocarrier surface through the hydrophobic PPO fraction [22].

Following intravenous injection to mice and rats, poloxamer- or poloxamine-coated sub-200 nm poly(phosphazene) [40], PLGA nanoparticles [41], and liposomes [42, 43] did not show prolonged circulation time as compared to the uncoated counterparts. This unexpected behaviour was ascribed to the desorption of the polymers from the nanocarrier surface [30] as well as to the polymer capacity to adsorb opsonins [44]. Indeed, the polymer composition has been found to affect the particle opsonisation as opsonins can associate with the hydrophobic polymer fraction that may be partially exposed on the particle surface [45, 46]. This possible effect can further contribute to the clearance of the polymer-coated nanocarriers.

For a given triblock polymer, it was found that both surface polymer density and coating layer thickness are affected by the particle size: smaller particles (below 100 nm) adsorb fewer polymer molecules per unit area than larger particles. Therefore, the polymer surface density decreases as the particle size decreases. Additionally, Pluronic adsorption on larger particles is relatively weaker than on smaller particles, which can affect the rate and extent of displacement of adsorbed polymers by blood components [47]. 

The surface adsorption efficiency and the stability of the polymer coating are strictly related to the polymer composition, namely, PO/EO molar ratio and PPO and PEO chain length [44]. 

Pluronic F-108 NF (poloxamer 338) has a bulkier central hydrophobic block and longer side hydrophilic arms (122 monomers of PEO; 56 monomers of PPO) as compared to Pluronic F-68 NF (76 monomers of PEO; 30 monomers of PPO). Accordingly, Pluronic F-108 NF forms more stable coating layers than Pluronic F-68 NF. In vivo, Pluronic F-68 NF-modified nanoparticles accumulate at 74% of the dose in the liver in 1 h, while the liver accumulation of Pluronic F-108 NF-modified nanoparticles was 67% [48].

#### 2.2.3. Dextran

Dextran is a polysaccharide largely used for biomedical applications including for the decoration of nanoparticulate drug delivery systems [49]. 

Dextran coating was found to bestow long circulating properties on liposomes [50]. Similarly to PEG, the steric brush of the dextran on the vesicle surface reduces the protein adsorption. This effect results in enhanced liposome stability in the blood [50], which depends on the density of dextran molecules. 

Interestingly, 70 kDa dextran coating was also found to reduce the burst of drug release from liposomes [50].

Dextran was used to coat superparamagnetic iron oxide nanoparticles for magnetic resonance imaging [51, 52]. Particles of 4 to 5 nm were coated with 20 to 30 dextran chains organized in “brush-like” structures, which reduced the removal from the bloodstream by Kupffer cells and splenic macrophages. The circulation half-life was prolonged to 3-4 hours [52]. The slight macrophage recognition of the dextran-coated superparamagnetic iron oxide nanoparticles was attributed to antidextran antibody opsonisation.

#### 2.2.4. Sialic Acid Derivatives to Mimic the Nature

Sialic acid derivatives received considerable interest as potential materials to confer stealth properties to nanoparticles for drug delivery applications. Sialic acid is a component of eukaryotic cell surface and plays an important role in preventing the removal of self-tissue by low level of complement activation through the alternative pathway. Desialylation of erythrocyte membranes results in reduction of factor H binding on their membrane that switches them from nonactivators to activators of the alternative complement pathway [53, 54]. Plasmatic circulating factor H adsorbed on bacteria or the surface of colloidal systems physiologically inhibits their complement-mediated destruction. This result is ascribable to factor H action as cofactor for the inactivation of the complement C3b factor and the alternative pathway convertase [55]. Therefore, factor H behaves as a dysopsonin. 

Surolia and Bachhawat demonstrated that liposomes coated with sialic acid derivatives are poorly recognised by the macrophages as they mimic the mammalian cell surface [56].

Stealth nanocarriers have been obtained using a variety of polysialic acid derivatives, including gangliosides [57–61], ganglioside derivatives, and glycophorin [62–64]. On the contrary, the coating with orosomucoid protein, a sialic acid rich protein, did not yield stealth poly(isobutylcyanoacrylate) nanoparticles. This effect was ascribed to the poor density of the sialic acid on the particle surface that does not allow for proper coating or to the inefficient conformation of the clustered glycans [65].

**Figure 2:**
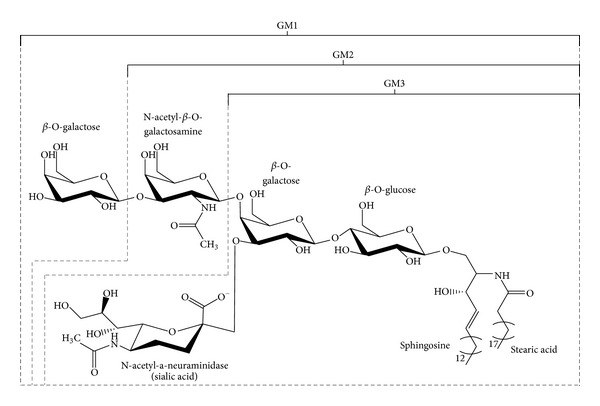
Chemical structure of the monosialoganglioside GM1.

The liposome coating with the monosialoganglioside GM1 (Figure 2), a brain-tissue-derived monosialoganglioside, was found to inhibit the alternative complement pathway by promoting the association of factor H to C3b factor on the vesicle surface [66]. In mice, the liposome decoration with 5–7 mol% of GM1 was found to increase the vesicle stability and inhibit the complement activation cascade, which resulted in prolonged permanence in the circulation [67]. As the molar ratio of GM1 in liposomes increases, the macrophage uptake inhibition increases up to 90% with 10 mol% GM1 [64].

Few studies postulated that the shielding of the negative charges of GM1 by the bulky, neutral hydrophilic sugar moieties is paramount to its stealth activity [58]. Nevertheless, other investigations showed that macromolecules bearing unshielded negative charges, namely, the ganglioside GM3, a sialic acid synthetic derivative, and a GM1 semisynthetic compound, increase the blood circulation time of sub-200 nm liposomes in mice [63]. Therefore, it can be concluded that the sterical organization of the ganglioside residues is primarily responsible for preventing the opsonisation of liposome containing glycolipids. 

Interestingly, studies performed with mice and rats showed that the gangliosides have a specie-specific activity. Indeed, the GM1 decoration was effective in mice while it did not have any beneficial effect on the circulation time of liposomes in rats [63].

#### 2.2.5. Zwitterionic Polymers

Zwitterionic phospholipid derivatives have been demonstrated to reduce the complement activation induced by liposomes [68].

Based on this evidence, synthetic zwitterionic polymers have been used to produce stealth drug delivery systems. These materials bind water molecules more strongly than polymers forming hydrogen bridges such as PEG. Furthermore, they provide electrostatically induced hydration [69] that decreases the rate of adsorption of proteins, cells, and bacteria on surfaces [70, 71]. Conversely than amphiphilic polymers, namely, PEG, that can partially insert itself in the lipid bilayer of liposomes [72, 73], zwitterionic polymers enhance the hydration of lipid polar group regions on the surface of liposomes and do not perturb the lipidic bilayer stability [74].

**Figure 3:**
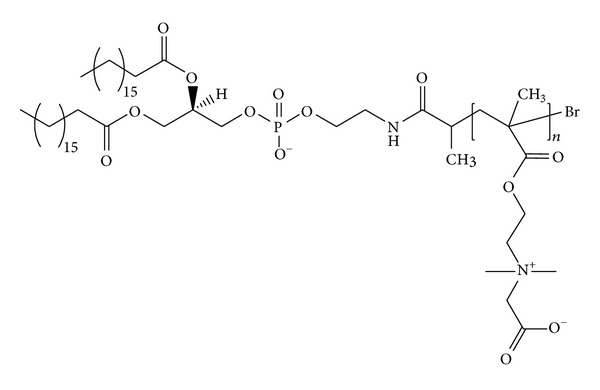
Chemical structure of poly(zwitterionic) poly(carboxybetaine)-DSPE derivative used to assemble poly-zwitterionic liposomes.

Liposomes coated with poly(zwitterionic) 2 and 5 kDa poly(carboxybetaine)-1,2-distearoyl-*sn*-glycero-3-phosphoethanolamine (poly(carboxybetaine)-DSPE) (Figure 3) possess similar stability of PEGylated liposomes. After 4 days of incubation at 37°C, no aggregation was observed. The enhanced hydration and fluidity of the liposome membrane provided by the poly(zwitterionic) component reduced its permeability and accounted for prolonged drug release as compared to the PEGylated counterparts. In vivo, poly(zwitterionic) polymer and PEG-coated liposomes showed similar pharmacokinetic profiles suggesting that the former may be used as an alternative to PEG [75]. 

Poly(carboxybetaine) is more chemically stable than PEG and has lower interactions with proteins over short and long time [76]. This material has been used to coat a variety of nanoparticles including silica [77], gold [78], iron oxide [79], PLGA [80], and hydrogel nanoparticles [81, 82]. In serum, the coated nanoparticles showed excellent stability to aggregation indicating that negligible opsonisation occurred as compared to other stealth particles [83]. This behaviour translates in exceptionally low unspecific cellular uptake. As an example, internalization of cross-linked poly(carboxybetaine)/iron oxide nanogels by HUVEC cells and macrophages was barely detectable [79].

#### 2.2.6. Polyglycerols

Polyglycerols (PGs) are biocompatible and flexible hydrophilic aliphatic polyether polyols, with an antifouling effect comparable to PEG [84]. By virtue of their multivalency that allows for the conjugation of targeting agents, drugs, labels, and physical modifiers [85], these polymers have been extensively studied as drug carriers.

Liposomes decorated with PGs exhibit extended blood circulation time and decreased uptake by liver and spleen [86].

Self-assembled monolayers (SAMs) of dendritic PGs were deposited on gold surface through a disulfide linker group (thioctic acid). Surface Plasmon resonance (SPR) measurements showed that PGs monolayers efficiently prevent the adsorption of proteins. It was concluded that dendritic PGs behave as antiopsonic materials because they combine the characteristic structural features of several protein-resistant materials: flexible aliphatic polyether structure, hydrophilic surface groups, and a highly branched architecture [84]. The inhibition of protein adsorption of hyperbranched polyglycerol was more efficient than linear PEG of similar molecular weight [87] and dextran. Furthermore, PGs have enhanced resistance to heat and oxidative stress as compared to PEG, which makes them potential candidates for biomedical applications [84].

#### 2.2.7. Polyacrylic and Polyvinyl Polymers

Synthetic polyacrylic and polyvinyl polymers bearing hydrophobic moieties have been prepared to coat liposomes. The hydrophobic function allows for the polymer anchoring on the particle surface.

Palmitoyl- or phosphatidylethanolamine- (PE-) terminated derivatives of poly(acryl amide) (PAA), poly(vinyl pyrrolidone) (PVP), and poly(acryloyl morpholine) (PAcM) have been found to exert comparable stealth effects on liposomes in vivo. This behaviour depends on the length of the hydrophobic alkyl function, the polymer molecular weight, and its surface density [88, 89].

Comparative studies performed with palmitoyl-or PE-functionalized 6–8 kDa PAA, PVP, and PEG showed that the PEG derivative has slightly better performance as compared to the other polymers. Macromolecules containing shorter hydrophobic moieties than palmitoyl- or phosphatidylethanolamine-, namely, dodecyl alkyl chains, or higher polymer molecular weight (12–15 kDa) showed a lower effect on circulation time of liposomes. Short hydrophobic moieties cannot efficiently anchor the polymer on the liposome surface as the energy of the polymeric chain motion is higher than the energy of the anchoring alkyl chain interaction with the liposomal phospholipid bilayer [88, 90]. The higher the polymer molecular weight, the higher the free energy of the exposed polymer chains. Therefore, the polymer can detach in vivo inducing liposome opsonisation and removal by the RES [91].

The layer thickness of poly(vinyl alcohol)s (6, 9, and 20 kDa PVA) derivatized with C_16_H_33_–S– as hydrophobic anchor (PVA-R) on the liposome surface was directly proportional to the polymer molecular weight and to the concentration of the polymer solution used for the coating process. Furthermore, it was found that the PVA-R density on the liposome surface increased as the molecular weight of the polymer decreased. The PVA-R on liposomes was not detached by dilution or in presence of serum while preventing the adsorption of plasma proteins. In vivo the PVA-R-coated liposomes showed prolonged permanence in the circulation, which increased as the PVA molecular weight increased. The circulation time of liposomes coated with 1.3% mol of 20 kDa PVA-R was comparable to that of liposomes coated with 8% mol of 2 kDa PEG-1,2-distearoyl-*sn*-glycero-3-phosphoethanolamine (PEG-DSPE). Detailed investigations showed that the increased permanence in the bloodstream was strictly related to the PVA-R stability on the liposome surface that was higher compared to PEG-DSPE [92].

### 2.3. Surface Requirements to Set Up Long Circulating Nanocarriers

The capacity of hydrophilic polymers to repel proteins is strictly related to the polymer composition, polymer molecular weight, density on the carrier surface, thickness of the coating, conformation, flexibility, and architecture of the chains. Furthermore, this capacity depends also on the physicochemical properties of the anchoring moieties that allow for the attachment of the polymer on the particle surface.

#### 2.3.1. Architecture and Molecular Weight of PEG Derivatives

The length of the polymer chains on stealth particle surface must exceed the range of the van der Waals attraction forces with soluble proteins in the bulk and phagocytic cells [93]. In the case of PEG, 2 kDa molecular weight is considered the lower threshold to guarantee macrophage avoidance. As the polymer molecular weight increases, the blood circulation half-life of the PEGylated particles increases [34, 94]. A study carried out with nanoparticles assembled using PEG-PLA block copolymer demonstrated that the 5 kDa PEG has the maximal capacity to reduce protein adsorption that yields to the uptake by phagocytic cells [33, 95].

High sensitivity differential scanning calorimetry was used to evaluate the effect of PEG size and acyl chain length of the PEG-phospholipid conjugate on the physical stability of liposomes [96]. The study was carried out with liposomes obtained using PEG-dipalmitoyl phosphatidylethanolamine (PEG-DPPE) and dipalmitoyl phosphatidylcholine (DPPC). A mixed lamellar/micellar phase was obtained with compositions containing more than 7% mol of 1–3 kDa PEG-DPPE while the complete conversion to micelles was achieved above 17% mol of PEG-DPPE. High molecular weight PEG-DPPE derivatives (12 kDa PEG-DPPE) could not be incorporated in the DPPC bilayer at all concentrations. The 5 kDa PEG-DPPE, which has an intermediate molecular weight, was partially miscible with DPPC at concentrations below 7% mol. Phase separation occurred above 7% mol 5 kDa PEG-DPPE while above 11% transition to micellar state was observed together with phase separation. In conclusion, stable stealth liposomes can be obtained with low ratio of 3–5 kDa PEG-DPPE. 

Concerning the hydrophobic anchoring moiety, longer alkyl chains than DPPE yielded unstable liposomes. PEG-DSPE embedded in a liposome distearoyl phosphatidylcholine (DSPC) bilayer promoted the phase separation even at low PEG-DSPE molar ratio (5%). This is ascribable to the steric restriction of the DSPE moiety within the bilayer due to high van der Waals cohesive forces that limit its mobility. This enhances dramatically the PEG chain/chain interactions that result in high mixing energy and favour demixing of the PEG-DSPE accompanied by structural rearrangements of the bilayer. Lipid phase separation generates domains on the liposome surface with low PEG-DSPE density that yields inhomogeneous PEG coating and poor sterical stability with rapid opsonin-mediated clearance. The phase separation would also lead to the leakage of encapsulated drug. On the other hand, short phospholipid alkyl chains, namely, PEG-dimyristoyl phosphatidylethanolamine (PEG-DMPE), embedded in liposome dimyristoyl phosphatidylcholine (DMPC) bilayer slightly delayed the formation of mixed lamellae/micelles at higher PEG-DMPE molar ratio (above 10%) than PEG-DPPE. The extent of demixing of PEG-phospholipid from bilayers decreases as the phospholipid alkyl chain decreases in the order of C18:0 > C16:0 > C14:0.

#### 2.3.2. PEG Density

The polymer density on the nanocarrier surface is as much relevant as polymer molecular weight. Few authors showed that the high polymer surface density can compensate the low polymer molecular weight in obtaining stealth particles [25, 95, 97]. Vittaz et al. investigated complement consumption of PEGylated PLA nanoparticles. The authors concluded that a distance between two chains of 2 kDa PEG of 2.2 nm corresponding to 0.2 PEG molecules/nm^2^ could achieve efficient 100 nm particle coating with minimum complement consumption [98]. Studies carried out using human phagocytes demonstrated that a distance of 1.4 nm between 5 kDa-PEG chains optimally yielded stealth 190–270 nm PEG-PLA nanoparticles [33]. However, it is worth to note that the polymer density threshold depends on a number of parameters, including particle size and surface curvature. 

Investigations carried out by decorating gold-coated silica particles with 750 and 2000 Da methoxy-PEG suggested that a polymer density of 0.5 chain/nm^2^ is a critical threshold to prevent the adsorption of plasma proteins [99].

Low complement consumption was observed in the case of 1.5 kDa PEG-stearate-coated 26 nm nanocapsules. The protein repulsion was found to depend on the polymer density rather than the polymer chain length [25, 100]. The nanocapsule surface covered by one PEG 1.5 kDa-stearate molecule was estimated to be about 2.8 nm^2^, corresponding to about 1.7 nm distance between two PEG chains, which is in fair agreement with the results described above. As a result of the low opsonisation and complement consumption, these nanoparticles displayed prolonged residence time in the blood with 20% of the dose still present in the blood 24 h after injection [101].

The homogeneous surface polymer coating is, together with the polymer density, a key parameter to obtain stealth particles. A study showed that 30% of PEGylated polystyrene nanoparticles underwent phagocytosis as a consequence of the inhomogeneous physical adsorption of the polymer on the particle surface [102].

#### 2.3.3. Liposome Rigidity and Cholesterol Effect

Phospholipid membrane rigidity is paramount to produce liposomes with stealth properties as well as to prevent rapid drug release. 

Decreased rigidity due to the use of phospholipids with low melting temperature (Tm) for the preparation of liposomal formulation can lead to drug leakage and opsonin adsorption.

The liposome membrane rigidity, homogeneity, and stability can be optimised by selecting phospholipids with proper Tm and by introducing cholesterol in the phospholipid bilayer. A minimum content of 30% mol cholesterol ratio is required to prevent the formation of phase separated lamellas and mixed micelles. It also reduces the leakage of encapsulated drug from liposomes [42, 103] and decreases the interaction of liposome surface with plasma components [96, 104].

#### 2.3.4. Surface Polymer Conformation

The polymer chain conformation on the particle surface plays a critical role in conferring improved stealth properties to nanocarriers. 

It was found that the optimal surface coverage to confer adequate stealth properties is the one that allows for a polymer chain conformation in between the “mushroom” and “brush” configurations. In this specific condition most of the chains are in a slightly constricted configuration, at a density to ensure no uncoated gaps on the particle surface. It is conceivable that predominant brush-like PEG configurations would sterically suppress the deposition of large proteins such as C3 convertase [25]. However, even when PEG is in the brush-like conformation on the surface of nanoparticles, its capacity to prohibit the protein adsorption on the surface is again affected by the obstruction capacity of the protecting layer. Small molecules can, in fact, slide in between the polymeric chains. For such a reason, Papisov et al. [105] highlighted the influence of (i) brush density, (ii) brush rigidity, (iii) brush molecular length, (iv) substrate size, and (v) cooperative character of interaction on steric repulsion and obstruction. 

The polymer chains conformation is dictated by the distance of the anchorage site of two polymer chains (*D*) and by the gyration radius of the polymer known as Flory radius (*R*_*g*_ = *αn* ^3/5^, where *n* is the number of monomers per polymer chain and **α** is the length of one monomer in angstroms which corresponds to 3.5 Å for PEG) [106]. The *R*_*g*_ of 2 kDa PEG is approximately 5.6 nm, which can be compressed depending on the surface grafting density. At low surface density, the PEG chains have higher mobility. In the case of *R*_*g*_ < *D* < 2*R*_*g*_ the polymer chain conformation corresponds to an intermingled “mushroom” configuration. This conformation allows the polymer chain for closer interactions to the surface of the particle and formation of gaps in the PEG protective layer that yields nanoparticle opsonisation [107]. High PEG density results in *D* ~ *R*_*g*_ and limited polymer chain motion that yields the transition from mushroom-like to mushroom/brush conformation. When *D* ≪ *R*_*g*_, the polymer chains convert to a brush-like conformation. The resulting low PEG chain mobility and flexibility reduces the ability of the polymer to repulse opsonins [23]. The polymer chain movement, due to its high flexibility and mobility, reduces both of the accessible surface of the nanoparticles and the interaction of the polymer with the cryptic pockets of the opsonins [108]. 

Studies performed with 100 nm liposomes coated with 2 kDa PEG-DSPE showed that below 4% PEG-DSPE molar ratio, the PEG chains were arranged in a mushroom conformation while a brush conformation was obtained above 8% PEG-DSPE molar ratio [109].

#### 2.3.5. Polymeric Corona Thickness

PEG layer thickness is paramount to obtain stealth nanoparticles. The minimum coating layer thickness required to guarantee efficient particle coating depends on a number of parameters including the potential absorbable proteins and the nanocarrier size [110]. 

Studies have shown that a minimum effective hydrodynamic layer thickness is about 5% of the particle diameter [111]. Moghimi et al. demonstrated that efficient protection of 60–200 nm polystyrene particles from complement activation and protein adsorption can be obtained with 4 kDa PEG that provides for a coating thickness of 5 nm [17].

The thickness of the polymer coating depends on the polymer chemical composition. In aqueous medium, PEG can provide for a maximum thickness corresponding to its full chain length. For copolymer such as poloxamers and poloxamines instead the thickness is linearly related to the number of EO monomers since only this function of the polymer can extend outward from the nanocarrier surface [93].

A hydrophilic polymer can provide for a surface coating thickness of *h*_*c*_ = *aN*(*a*/*D*)^1/*v*^, where *N* is the degree of polymerization, *a* is the size of the monomer, and *D* is the mean distance between grafting points [112]. For a good solvent the exponent is 3/5. 

In general, proper particle stabilization is achieved when *A*(*b*/*h*_*c*_) < *T* where *T* = temperature, *A* = Hamaker constant, and *b* = particle radius. As *A*/*T* is typically in the order of 1/10, a coating with a thickness corresponding to 10% of the particle diameter is conventionally considered adequate to provide for efficient steric stability [23].

#### 2.3.6. Polymer Flexibility

Studies have demonstrated that polymer chain mobility is required for repelling proteins from polymer chains on particle surface yielding stealth nanocarrier [113]. Accordingly, the lower complement activation of PEG as compared to dextran can be explained on the basis of polymer chain flexibility. In a CH50 assay, an in vitro haemolytic complement consumption assay, 10% complement activation was obtained with 20 cm^2^of 5 kDa dextran coated and 120 cm^2^ 5 kDa PEG-coated polycaprolactone nanoparticles [114]. The results normalized by the particle surface area show that the PEG coated particle surface induces a lower complement activation as compared to the dextran-coated surface. This is due to continuous change of the well-hydrated PEG chain conformation that reduces the exposure of fixation sites for complement proteins. The rapid movement of the flexible chains allows for the polymer to occupy a high number of possible conformations and leads to a temporary squeezing out of water molecules, making the surface impermeable for other solutes such as plasma proteins [108]. Therefore, the water cloud surrounding the PEG chains confers an interfacial free energy on the particle surface that protects the nanocarriers from opsonisation and recognition by macrophages.

#### 2.3.7. Amphiphilic Polymer Architecture

The coating polymer conformation on the nanocarrier surface is strongly affected by the polymer architecture which influences the plasma protein adsorption and interactions with cells. 

Nanoparticles obtained with multiblock (PLA-PEG-PLA)_n_ copolymers were found to adsorb higher amounts of proteins compared to nanoparticles obtained with polyethylene-glycol-grafted poly-(D,L) lactide (PEG-g-PLA) [115]. The low protein adsorption on PEG-g-PLA nanoparticles was ascribed to a higher surface PEG density. Similarly, nanoparticles obtained with copolymers with a PCL backbone and PEO grafts (PCL-g-PEO) were more effective in preventing protein adsorption as compared to PEO-b-PCL diblock copolymer nanoparticles [116]. 

The PEG attached through both terminal groups to the nanoparticle surface formed a single-turned-coil arrangement, which was found to provide compact conformational structures that endowed particles with high resistance against blood protein adsorption [117].

The effect of linear and branched PEGs on stealth properties of nanocarriers was also investigated by using liposomes decorated with PEG-PE and PEG_2_-PE. PEG_2_-PE was more efficient in improving the blood circulation time than PEG-PE at a low content (3% mol), whereas at high molar ratio (7% mol) their effect on liposome blood clearance is almost identical. At higher ratio of protecting polymer (7% mol), even PEG-PE can provide complete coating of the liposome surface that does not take place at low molar PEG-PE ratio [108].

### 2.4. Controversial Effect of Polymer Coating

Many studies have demonstrated that the particle opsonisation can be reduced by surface coating with hydrophilic flexible polymers and mathematical elaborations have been developed to describe this effect. However, it should be noted that several controversial results have been reported in the literature.

In vitro studies showed that stealth vesicles obtained by PEG coating can associate with a pool of opsonic proteins of serum and plasma such as components of the complement system and immunoglobulins. Nevertheless, it was not clear if the protein interaction occurred with the exposed or internal part of the coating polymer [14, 29, 33, 60, 118–124]. In vivo, 2.5–10% of the dose of PEG-coated vesicles and nanoparticles has been found to dispose in the liver and spleen in the first hour after intravenous administration [125–130]. The limited removal of stealth particles from the bloodstream seems to indicate that a small amount of specific opsonic proteins can target PEG-coated nanocarriers [124]. This hypothesis is supported by the evidence that low doses (20 nmol/kg body weight) of PEGylated liposomes are rapidly cleared by macrophages, while the cleared dose fraction decreases as the amount of the injected PEG-coated liposomes increased [125–127].

Stealth nanocarriers were found to display long circulation profiles even after extensive opsonisation. A typical example is Doxil, the PEGylated doxorubicin loaded liposome formulation, which is efficiently opsonised by the C3b factor and activates the complement. Nonetheless, Doxil presents a biphasic circulation half-life with prolonged permanence in the circulation [21].

Overall these data show that the stealth behaviour of long circulating nanocarriers is a very complex mechanism and it cannot be reduced to the simple opsonin repulsion underlining some additional and relevant effects operated by the steric coating on the nanocarrier surface.

#### 2.4.1. PEG Induced Complement Activation

PEG coating on one side reduces the opsonisation process, while on the other can induce the complement activation that is involved in the nanoparticle removal. Liposomes are a typical example of the double effect of particle PEGylation.

Liposomes with low surface charge obtained with saturated phospholipids and high cholesterol content, which endows rigid and uniform bilayer without surface defects, are poorly prone to opsonisation and structural destabilisation by C3 adsorption [121, 128, 131, 132]. On the contrary, negatively charged and flexible liposomes undergo rapid opsonisation and phagocytosis. The incorporation of 5–7.5 mol% of PEG 2 kDa-DSPE into the bilayer of anionic liposomes formed by egg phosphatidyl-choline, cholesterol, and cardiolipin (35 : 45 : 20 mole ratio) was found to dramatically reduce the complement activation of these vesicles. However, the degree of complement activation also depended on the liposomes concentration. Indeed, in vitro studies showed that 15 mM PEGylated liposomes concentration induced 40% complement consumption [133]. 

Studies carried out with Doxil showed that 0.4 mg/mL of PEGylated liposomes elicited the rapid complement activation and generate the soluble terminal complement complex (SC5b-9) in 7 out of 10 human sera [134]. These results underline the individual effect of PEGylated liposomes on the complement activation. 

The complement activation by PEGylated liposomes was found to be responsible for several side effects. In pigs Doxil was demonstrated to activate the complement through both the C1q-dependent classical and the alternative complement activation pathways [135], which was responsible for the cardiopulmonary distress [136].

In few cases, a transient in vivo response was observed in rabbits as a drop in the systemic arterial pressure at 10 min after liposome injection which is typical of the complement activation [137]. On the contrary, no complement activation after PEGylated liposome administration was evidenced by the in vitro assay. These evidences highlight that in vitro complement activation tests should be carefully evaluated for what concerns their sensitivity and response threshold in order to obtain results that can be correlated with the in vivo data. 

Studies performed with PEGylated polymeric nanoparticles confirmed that PEG-coated systems can induce the complement activation regardless of the PEG chain length and surface density. The complement activation was inversely correlated with the PEG molecular weight suggesting that steric hindrance on the particle surface due to the polymer coating reduces the approach and association of large proteins such as the C3 convertase [97, 138].

Studies carried out using PEGylated erythrocytes showed that the complement activation may be mediated by anti-PEG IgG and IgM [139].

Anti-PEG IgM elicited by a first administration of PEGylated liposome forms immunocomplexes with the second dose of liposomes [140]. These complexes activate the complement and convert the C3 component into C3b. The complex formed by C3b with other complement components is involved in the antibody-mediated complement activation pathway [134, 141] that yields C3b fragmentation to iC3b operated by factors H and I. iC3b is a proteolytically inactive product of the complement fragment C3b that can still opsonise. However, it cannot participate in the complement cascade since it does not associate with factor B, a component of the alternative activation pathway in the early stage of the activation. The generation of iC3b prevents the amplification of the complement cascade. Overall the PEG molecules on the liposome surface do not interfere with production of opsonic components from the C3 component. 

Complement activation has been suggested to account for the clearance of PEGylated liposomes by the macrophage uptake of the RES [142].

Furthermore, the extent of the accelerated blood clearance (ABC) of PEGylated liposomes is inversely proportional to the dose probably because of the saturation of the mononuclear phagocytic system [143]. 

#### 2.4.2. Poloxamine Induced Complement Activation

Similarly to PEG, Poloxamines and Poloxamers have been extensively used to endow nanocarriers with stealth properties. Nonetheless, even these materials have been found to activate the complement to some extent thus reducing the beneficial effect on particle opsonisation.

Poloxamine-908-coated polystyrene nanoparticles were found to activate the complement through a complicated pathway. The adsorbed poloxamine-908 on the polystyrene nanoparticles rearranges from flat mushroom-like to brush-like conformation as the density of the polymer on the particle surface increases. As the polymer packs on particle surface, the surface area occupied by poloxamine decreases from 45 to 15 nm^2^/poloxamine chain. The intermediate mushroom-brush poloxamine conformation induced remarkable complement activation that decreased when the polymer rearranged to a brush-like structure. Uncoated nanoparticles and particles coated with poloxamine in the mushroom-like conformation promote surface association of the C1q fragment of the complement protein C1 and activate the complement through the classical pathway. Naked and poloxamine-coated nanoparticles in the mushroom and mushroom-brush conformation also activate the complement through the alternative pathway by covalent conjugation of properdin to poloxamine and the C3 component adsorption. Conversely, particles coated with poloxamine in the mushroom-brush and fully brush conformation activate the complement via the lectin pathway, which involves the opsonisation of mannose-binding lectin protein (MBL) and/or ficolins. This complement activation pathway was attributed to the structural similarities between the EO monomers of poloxamine and a region of D-mannose [144]. The brush-like conformation minimizes the MBL and ficolin binding to PEG backbone and consequently reduces the complement activation via the lectin pathway [145].

Thus, the conformation and the mobility of surface projected PEO chains of poloxamine on nanoparticles are paramount to modulate the complement activation pathway [146].

### 2.5. “Long Circulation” Revealed

PEG-and poloxamine-coated nanocarriers have been demonstrated to undergo immunoglobulin, fibronectin, and apolipoprotein association [14, 29, 33, 118, 122–124, 147] as well as C3 opsonisation that mediates the biorecognition by macrophages through specific complement receptors (CR1 and CR3, CD11b/CD18) [18]. However, these systems possess long-lasting profiles in blood [148]. The prolonged circulation in the bloodstream is due to the steric hindrance of the surface polymers [134] that prevents the macrophage approach [124]. Furthermore, the C3b adsorbed on the polymer corona of the particle surface can be proteolytically degraded to fragments that by assembling with other cofactors inhibit the recognition by the macrophage receptors [149]. The factor C3bn of the complement adsorbed on PEG-coated liposomes may also bind CR1 receptor associated with the erythrocytes membrane, which can also explain the prolonged circulation time of PEGylated liposomes [150].

The steric shielding effect conveyed by polymer coating on long circulation properties of stealth nanocarriers was demonstrated by Moghimi using poloxamine-908-coated particles. These particles, incubated with serum obtained from a poloxamine-908 preinjected animal, showed a higher protein adsorption as compared to particles incubated with serum obtained from animals that were not preexposed to poloxamine. The protein-coated nanoparticles showed similar pharmacokinetic profiles when administered to animals never exposed to poloxamine. This evidence reinforces the explanation that the improved circulation time of stealth nanoparticles is not solely ascribable to reduced protein adsorption on particle surface [151] which surely takes place for sterically stabilized nanocarriers. Improved circulation time can be mainly attributable to the prohibited biorecognition of the adsorbed opsonic proteins by the macrophages.

### 2.6. Nanocarrier Coating with Hydrophilic Polymers: Physical and Chemical Strategies

Sterically protective polymer can be physically or chemically conjugated to the nanocarrier surface. Physically conjugation involves the hydrophobic adsorption of polymer fragments on the particle surface while the chemical conjugation is obtained by chemical reaction of polymers with surface functions to yield covalent bonds. 

So far a variety of protocols have been set up to conjugate PEG to small molecules and biologically active proteins. These methods have been translated to obtain stealth nanoparticles with other materials [152, 153].

#### 2.6.1. Physical Coating of Polymeric Nanoparticles and Liposomes

Surface PEG coating of PLGA nanoparticles was carried out using 2 kDa PEG-DSPE as emulsifier during oil-in-water microemulsion nanoparticle preparation. The process allows for the embedding of the PEG-DSPE phospholipid fraction in the PLGA matrix by hydrophobic interactions, whereas the hydrophilic PEG chain extends outward the nanoparticle surface, forming a polymeric brush that stabilizes the system. Drug loaded 120 nm PEGylated PLGA nanoparticles were successfully used for the treatment of a cystic fibrosis murine model by intranasal administration [154].

An original multistep technique for physical PEGylation of doxorubicin loaded PLGA nanoparticles involves the surface adsorption of palmitate-avidin on the particles through the avidin alkyl chain anchor during the particle preparation by emulsion. The avidinated particles are subsequently PEGylated by exposure to PEG-biotin. The particle coating with 5 and 10 kDa PEG reduced protein adsorption by 50, and 75%, respectively, compared to the non-PEGylated PLGA nanoparticles. Approximately 3% of the initial dose of the doxorubicin loaded nanoparticles intravenously administered was detected in the serum after 48 hours from administration. This corresponds to a twofold residual doxorubicin plasma concentration as compared to that obtained with non-PEGylated particles [155].

Protective PEG layer on liposomes can be achieved through two very conventional strategies. 

**Figure 4:**
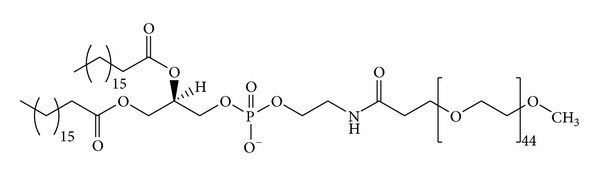
Structures of PEG-lipid conjugates used in preparing stealth liposomes. The derivative is obtained with a PEG chain of 45 monomers, corresponding to a molecular weight of approximately 2000 Da. PEG units are capped at the distal end with a methoxy group, and conjugated to a DSPE lipid.

In the first approach PEG is conjugated with a hydrophobic moiety (usually the residue of PE or a long chain fatty acid is reacted with methoxy-PEG-hydroxysuccinimide ester) [156, 157] (Figure 4). Subsequently a dry mixture film of phospholipids and the mPEG-PE is rehydrated to yield liposomes that spontaneously expose the PEG chains on their surface [158]. 

A second approach to coat liposomes with PEG is called the “postinsertion method” and consists in the conjugation of activated PEG to preformed liposomes. 

#### 2.6.2. Polymer Coating of Magnetic Iron Oxide Nanoparticles 

Specific coating protocols have been set up to produce stealth inorganic nanoparticles.

The incorporation of a polymer coating on the nanoparticle surface can be achieved either via “one-pot” methods, where the nanoparticles are coated by a polymer dissolved in the particle production mixture, or by “two-step” or “postproduction” method, where nanoparticles are first generated and then coated with a polymer.

Magnetic nanoparticles coated with PEG-based copolymers have been prepared in one pot by Fe_3_O_4_ nucleation and growth. Poly(ethylene glycol) monomethyl ether-b-poly(glycerol monoacrylate) (PEG-b-PGA) was added to Fe^2+^/Fe^3+^ solutions and the coprecipitation of the iron ions was induced. The iron atoms on the nanoparticle surface were coordinated via the 1,2-diols of the PGA block, which resulted in particle stabilization [159].

Iron oxide nanoparticles stabilized by carboxyl coordination of the surface oxide molecules were prepared by high-temperature decomposition of tris(acetylacetonate) iron(III) [Fe(acac)_3_] in the presence of monocarboxyl-terminated PEG [160].

Postproduction iron oxide nanoparticle decoration was performed using silane-terminating PEG. The silane group strongly interact with the oxide on the nanoparticle surface [161]. PEGs derivatised with amino propyl trimethoxy silane (APTMS) or amino propyl triethoxy silane (APTES) were used.

Phosphonic acid-terminated poly(oligoethylene glycol acrylate) [poly(OEGA)] was grafted to iron oxide nanoparticles through the phosphonic acid end group that provide strong interaction with iron oxide nanoparticles. The poly(OEGA-) stabilized iron oxide nanoparticles showed significant stealth properties and exhibited low BSA adsorption (<30 mg g^−1^ nanoparticles) over a wide range of protein concentration (0.05 to 10 g L^−1^) [162].

Iron oxide nanoparticles synthesized by Fe(acac)_3_ decomposition in high-boiling organic solvents were postproduction PEGylated by the ligand exchange method. The nanoparticles produced with oleic acid, hexane, or trioctyl phosphine oxide (TOPO) coating were combined with PEG-silanes, PEG-PEI, PEG-PAMAM, PEG-fatty acid to allow for the coating exchange in aqueous medium [163–168].

Dopamine has been proposed as an alternative anchoring group to silane to coat magnetic nanoparticles. Dopamine has high affinity for the iron oxide and can be conjugated to PEG through the amino group. PEG-dopamine was used to displace the oleate/oleylamine coating on the particles produced by high-temperature decomposition of Fe(acac)_3_ thereby converting the particle surface from hydrophobic to hydrophilic according to a postproduction protocol [169].

“Growing from” approaches based on living radical polymerization techniques such as Atom-Transfer Radical-Polymerization (ATRP) and Reversible Addition-Fragmentation chain-Transfer (RAFT) polymerization have been largely investigated to coat preformed iron oxide nanoparticles with PEG copolymers. ATRP polymerization of PEG-methacrylate (PEG-MA) was performed in aqueous solvent after a silane initiator (4-(chloromethyl) phenyl trichlorosilane) immobilization on iron oxide nanoparticle surface. After poly(PEG-MA) grafting, the uptake of the nanoparticles by macrophages was reduced from 158 to less than 2 pg per cell confirming the excellent shielding capacity of this novel material [170].

Alternatively, the ATRP polymerization of the PEG-MA was performed according to a solvent-free protocol. The macroinitiator on the surface of the magnetic iron oxide nanoparticles was introduced by exchanging the surfactant (oleic acid) on the nanoparticle surface with 3-chloropropionic acid. The exchange made the nanoparticles soluble in PEG-MA that was then polymerized by ATRP. No difference in terms of capacity to evade macrophage uptake was detected when poly(PEG-MA-) coated iron oxide nanoparticles were prepared in water or by the solvent-free method [171].

Hyperbranched polyglycerol (HPG) has recently emerged as a biocompatible and resistant material to protein adsorption, which was ascribed to its hyperbranched nature [84]. HPG-grafted magnetic iron oxide nanoparticles have been prepared by surface-initiated anionic polymerization of glycidol. Iron oxide nanoparticles were first functionalized with 3-mercaptopropyltrimethoxysilane that, in the anionic form, promotes the ring opening polymerization of glycidol in toluene. A 13 wt% HPG coating was obtained by this procedure. The protein adsorption was very low and comparable to that of nanoparticles grafted with silanated methyloxy-PEG (MW = 750 Da) at a similar grafting density [172]. Glycidol polymerization can be also initiated by aluminium isopropoxide grafted to 6-hydroxycaproic acid coated iron oxide nanoparticles. The resulting 24 nm HPG-grafted nanoparticles are very stable in PBS and culture media and their uptake by macrophages was very low (<3 pg Fe/cell), over a 3-day contact time [173].

#### 2.6.3. Polymer Coating of Gold Nanoparticles 

Gold nanoparticles have been PEGylated according to “one-pot” methods. AuCl_3_^−^ in solution can in fact be reduced by the amino groups of the PEI block of poly(ethylenimine)-poly(ethylene glycol) block copolymer (PEI-b-PEG) [174].

Postproduction PEGylation strategies have relied mostly on the use of thiol (-SH) terminated PEGs because of the very high specific binding affinity of thiol groups to metal gold (S-Au bond energy = 47 kcal mol^−1^). Thiol-PEG can react in solution with gold nanoparticles providing colloidally stable and biocompatible gold nanoparticles [175].

Bidentate PEGs (PEG-thioctic acid and PEG-dihydrolipoic acid) conjugated on gold nanoparticle surface substantially improved the stability in biological media [176]. Gold nanoparticles PEGylated with thioctic-modified 5 kDa PEG were shown to perform better in vivo than gold nanoparticles coated with thiol-PEG since the latter can release the PEG by exchange with thiolated compounds in the body [177].

The in vivo performance of gold nanorods stabilized with thiol-PEG depends on the polymer molecular weight. Accordingly, stable nanorods for blood circulation were obtained with 5 and 10 kDa PEGs while smaller or larger PEGs were poorly flexible or bend into a mushroom-like configuration, respectively [34, 178].

The maximum achievable density of PEG chains on gold nanoparticles was 2.2 nm^2^ per chain, which is comparable to the hydrodynamic size of the mPEG-thiol molecule [179]. At saturation, the PEG molecules are so tightly packed that opsonins will be prevented from adsorbing on the coating layer thus prohibiting the binding to macrophage receptors.

Layer-by-layer (LBL) coating approaches relying on electrostatic interactions between polymer chains and gold nanoparticle surface have been investigated to build up a hydrophilic polymer corona on gold nanoparticles. The colloidal core of gold nanoparticles was coated with layers of poly(allylamine) (PAH) and poly-(styrenesulfonate) (PSS). F-HPMA, a hydrophilic terpolymer composed by 90% mol of N-(2-hydroxypropyl) methacrylamide, was then conjugated to the amino groups of PAH to yield core/shell multifunctional nanoparticles. The terpolymer provides a highly water-solvated corona layer that minimizes the opsonisation process and bestows remarkable stealth properties on nanoparticles. The multifunctional nanoparticles did not show a significant degree of adsorption on the macrophage membrane or internalization by the cells [180].

PEG was grafted on gold nanoparticle surface according to a process named physisorption. PEG-NH_2_ and 1,2-distearoyl-*sn*-glycero-3-phosphoethanolamine (DSPE) were conjugated to the backbone of polyglutamic acid (PGA) at 60% and 10% mol ratio with respect to the PGA monomers, respectively. Gold nanoparticle coating was achieved by exchanging the citrate adsorbed on gold particles, obtained by tetrachloroauric acid reduction, with the multifunctional polymer PGA-DSPE-mPEG. These functionalized colloidal systems showed high stability to aggregation over 48 hours of incubation in 50% fetal calf serum [181].

Polyethylene glycol-block-poly(2,*N,N*-dimethylamino) ethyl methacrylate (PEG-b-PAMA) was shown to improve the long-term stability of gold nanoparticles. The tertiary amino group of PAMA can strongly adsorb to the surface of gold nanoparticles even though the mechanism of immobilization is not clear yet. The alkylation of pendant amino groups along the polymer backbone seems to favour the interaction of the nitrogen atom with gold. The colloidal system was physically stable over 4 days of storage in 95% human serum [182].

Gold nanoshell can also be coated with a variety of polymers according to the same postproduction strategies reported for gold nanoparticles and nanorods. 

#### 2.6.4. Polymer Coating of Silica Nanoparticles

Silica nanoparticles possessing an organosilica core and a PEG shell were prepared according to a one-pot procedure. The process includes the co-hydrolysis and copolycondensation reactions of *ω*-methoxy-(polyethyleneoxy)propyltrimethoxysilane and hydroxymethyltriethoxysilane mixtures in the presence of sodium hydroxide and a surfactant [183].

Alternatively, silica nanoparticles were also PEGylated by a postproduction procedure by mesoporus silica nanoparticle reaction with PEG-silanes. It was reported that the PEG coating inhibits the nonspecific binding of human serum proteins to PEGylated silica nanoparticles. This is a guarantee if the molecular weight of the polymer is higher than 10 kDa and the polymer density (defined as wt% of the coating on the mesoporous silica nanoparticles) is 0.75 wt% and 0.075 wt% for PEG 10 kDa and PEG 20 kDa, respectively. The human serum albumin adsorption was only 2.5 wt% when PEGylated silica nanoparticles were tested compared to 18.7% for non-PEGylated nanoparticles [184].

PEG coating on silica nanoparticles can also be achieved via electrostatic adsorption of polyethyleneimine-polyethylene glycol (PEI-PEG) copolymer. The polymeric coating was stable and tightly associated with the particle surface by virtue of the strong electrostatic interactions between the polyamino backbone of the copolymer and the negatively charged silica surface. The PEI-PEG copolymer investigated had 34 PEG chains (5 kDa) per PEI chain. The efficiency of the PEG coating in preventing the adsorption of serum proteins on the nanoparticle surface was remarkably high. Protein adsorption was at the limit of sensitivity for X-ray photoelectron spectroscopy (XPS) detection and no aggregation was observed for the coated nanoparticles [185]. 

The synthesis of PEO on silica nanoparticles has also been performed resulting in a 40 wt% of grafted PEO. The method has been carried out first by a two-step conjugation process of prehydrolyzed 3-glycidoxypropyl trimethoxysilane and aluminium isopropoxide to the particle surface. The subsequent polymerization of ethylene oxide was carried out at 55°C. The density of the polymer chains was found to be strictly dependent on the conjugation efficiency of the metal alkoxide on the particle surface [186, 187].

## 3. Conclusions

The therapeutic advantages of nanotechnology-based drug delivery systems include improved drug bioavailability, extended duration of action, reduced frequency of administration, and lower systemic toxicity with beneficial effects on the patient acceptance. The medical management of malignancies has already benefited from the outcomes of few nanotechnology-based delivery systems. However, following intravenous administration, drug-loaded nanocarriers are rapidly opsonised by a variety of proteins, most of them belonging to the complement system, and undergo very rapid clearance via the MPS cells. 

In this paper, the main aspects of polymer coating technology applied to colloidal drug delivery systems have been reviewed. A number of studies and examples reported in the literature showing that stealthiness can be conferred to nanocarriers by a proper formulation design and predicated by precise physicochemical determinants have been detailed and critically discussed. 

The evidence reported in the literature shows that the residence time in the blood of nanocarriers can be prolonged by surface coating with neutral or zwitterionic polymers characterized by high hydrophilicity and high flexibility. Furthermore, the stealth character of the nanocarriers depends on the polymer organization on the particle surface, namely, density, thickness, and association stability. The beneficial effect of nanocarrier polymer coating in promoting stealth properties generates predominantly from the polymer ability to confer a physical barrier to the biorecognition of adsorbed opsonins by macrophages. On the other hand, the paper underlines that the components of the hydrated polymeric corona are not completely inert to the biological environment and these materials do not totally prohibit the protein opsonisation [124]. 

In conclusion, while many discoveries in the field of nanotechnology have allowed to clearly improve the performances of stealth nanocarriers, a significant amount of work needs to be done before these systems achieve the required level of safety for use in humans. Studies are required to fully profile at the molecular level the interactions of the nanocarriers with the biological environment and the MPS cell response that is triggered upon contact with a specific nanocarrier.
